# Application of Non-Invasive Cardiac Output Monitoring in Enhanced Recovery After Surgery for Patients With Acute Type A Aortic Dissection

**DOI:** 10.31083/RCM52101

**Published:** 2026-09-14

**Authors:** Jian Zheng, Xiangkun Xiao, Ziheng Ye, Lin Huang, Meng Rui, Yiwen Lin, Fuhua Qiu, Lei Sun, Qingsong Wu

**Affiliations:** ^1^Department of Pharmacy, The First Affiliated Hospital of Fujian Medical University, 350001 Fuzhou, Fujian, China; ^2^School of Health, Fujian Medical University, 350001 Fuzhou, Fujian, China; ^3^Department of Cardiovascular Surgery, Fujian Medical University Union Hospital, 350001 Fuzhou, Fujian, China; ^4^Cardiac Rehabilitation Department, Fujian Medical University Union Hospital, 350001 Fuzhou, Fujian, China

**Keywords:** aortic dissection, aorta surgery, cardiac output, enhanced recovery after surgery, treatment outcome

## Abstract

**Background:**

To assess the clinical value of non-invasive cardiac output monitoring (NICOM) within an enhanced recovery after surgery (ERAS) protocol for patients undergoing acute type A aortic dissection (ATAAD) repair.

**Methods:**

This study included 242 patients who underwent total arch replacement for ATAAD between January 2024 and June 2025. Patients were stratified into two groups based on postoperative NICOM use (NICOM group, n = 141; control group, n = 101). This study compared hemodynamic parameters, 6-minute walk test (6MWT) results, postoperative recovery, and complications.

**Results:**

Baseline characteristics were similar between groups. While 30-day mortality and ventilator time were comparable, the NICOM group demonstrated significantly superior outcomes inintensive care unit (ICU) stays [3.0 (2.0–5.0) vs. 5.0 (3.0–8.0) days; *p* < 0.001] and hospitalization time [16.0 (11.0–21.0) vs. 19.0 (14.0–23.0) days; *p* = 0.001]. The NICOM group also showed lower unadjusted rates of reintubation (1.4% vs. 7.9%; *p* = 0.029), ICU readmission (2.1% vs. 8.9%; *p* = 0.036), shock (0.7% vs. 5.9%; *p* = 0.045), and acute heart failure (2.1% vs. 9.9%; *p* = 0.018), as well as a longer 6MWT distance at discharge [488.0 (433.0–532.0) vs. 457.0 (410.0–521.0) meters; *p* = 0.02]. However, after applying Bonferroni correction for multiple comparisons, only ICU stay and hospitalization time remained statistically significant; the differences in re‑intubation, ICU readmission, shock, acute heart failure, and 6MWT did not meet the corrected significance threshold.

**Conclusion:**

Integrating NICOM into the postoperative care of patients with ATAAD provides reliable, continuous hemodynamic data that facilitate precise, individualized management. This approach stabilizes circulation, reduces key complications, accelerates recovery, and improves perioperative outcomes, underscoring the vital role of this method in implementing ERAS protocols in cardiac surgery.

## 1. Introduction

Acute type A aortic dissection (ATAAD) is a catastrophic cardiovascular emergency characterized by abrupt onset and high mortality rates, for which surgical intervention remains the only definitive treatment [1]. Total arch replacement is a commonly employed surgical procedure for ATAAD. However, this operation is associated with significant trauma, prolonged duration, and frequent postoperative complications such as hemodynamic instability and multiple organ dysfunction, which severely impede patient recovery and quality of life [2,3,4]. In recent years, the enhanced recovery after surgery (ERAS) protocol has been increasingly adopted in cardiac surgery. Its core principle is to minimize surgical stress response and accelerate patient recovery through evidence-based, multimodal perioperative management strategies [5,6,7,8].

Non-invasive cardiac output monitoring (NICOM) technology, utilizing electrical biosensing technology, enables the non-invasive, continuous, and real-time monitoring of key hemodynamic parameters including cardiac output and stroke volume, providing crucial data for clinical assessment of circulatory status [9,10,11]. Nevertheless, the value of NICOM within the context of ERAS management for post-operative ATAAD patients remains underexplored and lacks systematic evaluation. And the existing pulse index continuous cardiac output (PICCO) and esophageal Doppler monitoring methods are invasive. Therefore, this study aims to investigate the efficacy of implementing NICOM in the postoperative management of ATAAD patients and to assess its impact on recovery and clinical outcomes, thereby providing evidence for the deeper integration of the ERAS concept in cardiac surgery.

## 2. Materials and Methods

### 2.1 Study Population

We retrospectively enrolled patients who underwent total arch replacement for ATAAD at our institution between January 2024 and June 2025. The diagnosis of ATAAD was confirmed in all patients via computed tomography angiography (CTA) of thoracoabdominal aorta. Included patients underwent emergency total arch replacement and were aged ≥18 years. The exclusion criteria were as follows: (1) pre-existing severe arrhythmias (persistent atrial fibrillation, frequent ventricular premature contractions) that could potentially compromise the accuracy and stability of NICOM data; (2) preoperative or intraoperative occurrence of severe cardiogenic shock or cardiac arrest requiring advanced life support such as extracorporeal membrane oxygenation or intra-aortic balloon pump; (3) requirement for emergency re-sternotomy due to active bleeding or severe coagulopathy; (4) presence of significant skin lesions, infections, or deformities at electrode placement sites preventing proper electrode attachment; (5) inability to maintain stable vital signs despite the use of vasopressors or inotropic agents; (6) incomplete clinical data (missing more than 3 items) or loss to follow-up (Fig. 1).

**Fig. 1.**
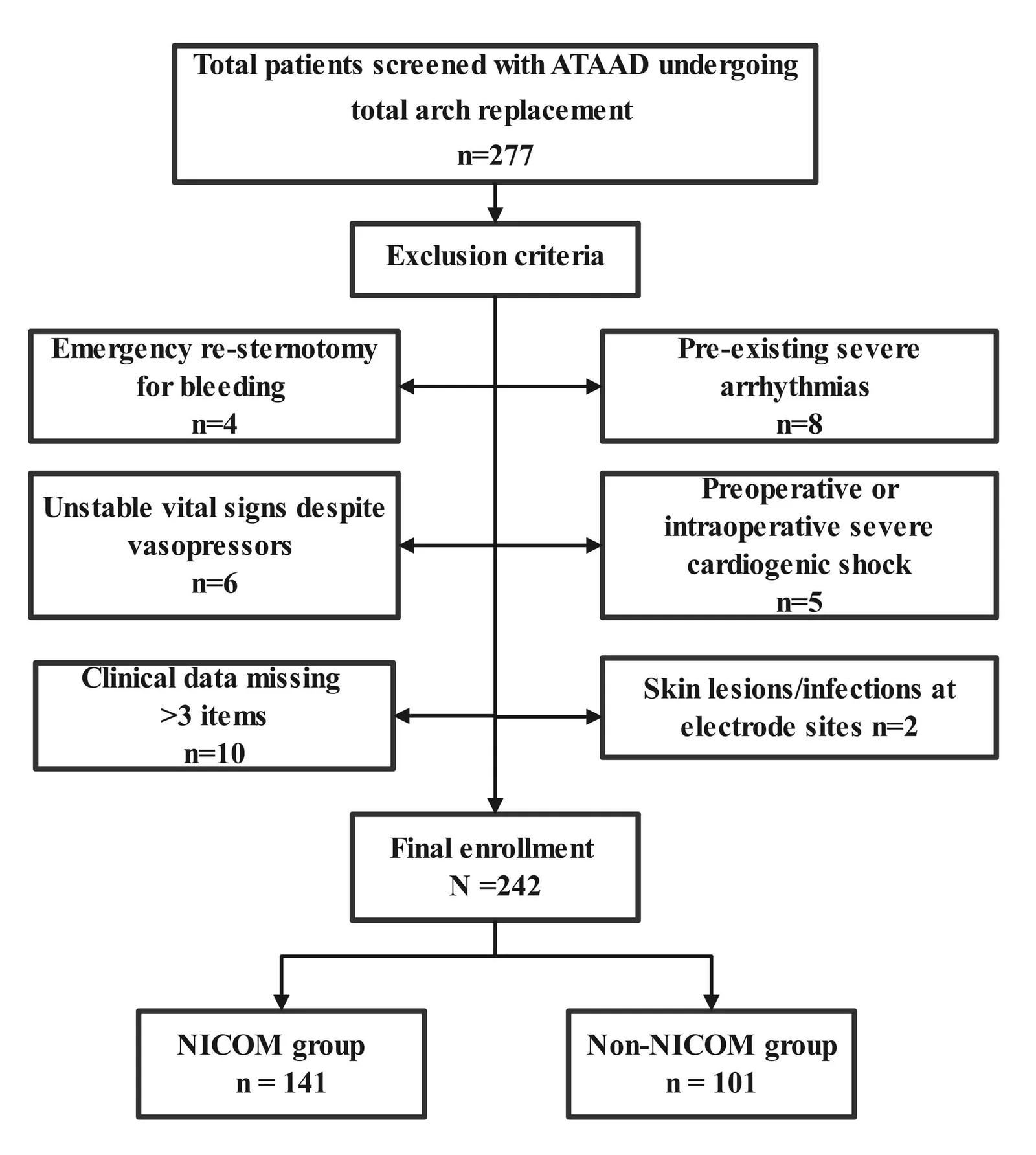
**Flowchart of the study cohort**. ATAAD, Acute type A aortic dissection; NICOM, Non-invasive cardiac output monitoring.

30-day mortality defined as death from any cause within 30 days after surgery, or during the same hospitalization if beyond 30 days. Mechanical ventilation time defined as total duration (hours) of invasive ventilator support from intensive care unit (ICU) admission to first successful extubation (≥48 hours without reintubation). Reintubation defined as need for endotracheal intubation within 72 hours after planned extubation. Acute heart failure defined as clinical signs of heart failure plus need for inotropes or vasodilators plus pulmonary congestion on chest X-ray and/or ≥10% decrease in left ventricular ejection fraction (LVEF) from baseline.

### 2.2 Study Parameters

The studied parameters encompassed patient baseline and preoperative characteristics, surgical data, and postoperative outcomes. Baseline and preoperative characteristics included sex, age, body mass index, history of coronary artery disease, hypertension, diabetes mellitus, LVEF, and serological test results. Surgical parameters consisted of operative time, cardiopulmonary bypass (CPB) time, aortic cross-clamp time, selective cerebral perfusion (SCP) time, and intraoperative blood loss. Postoperative metrics included duration of mechanical ventilation, length of ICU stay, hospitalization time, rates of reintubation and ICU readmission, and 30-day mortality. Major adverse outcomes recorded were acute kidney injury, acute respiratory distress syndrome (ARDS), acute heart failure, shock, cerebral hemorrhage, cerebral infarction, gastrointestinal bleeding, sepsis, spinal cord injury, and multiple organ dysfunction syndrome (MODS). Additionally, postoperative NICOM (ICG1000, Shenzhen Meilin Medical Technology Co., Ltd., Shenzhen, Guangdong, China) monitoring data and the pre-discharge 6-minute walk distance were collected.

This study was conducted in accordance with the principles of the Declaration of Helsinki and was approved by the Ethics Committee of Fujian Medical University Union Hospital (2025KY711). In compliance with national guidelines, written informed consent was waived for this retrospective review of patient data.

### 2.3 Statistical Analysis

Data analysis was performed using SPSS software (version 27.0, IBM Corp., Armonk, NY, USA). Continuous variables are presented as mean ± standard deviation if normally distributed, or as median and interquartile range [M (Q1, Q3)] otherwise. Comparisons between groups for continuous variables were conducted using the independent samples *t*-test or the Mann-Whitney U test, as appropriate. Categorical variables are expressed as numbers and percentages, and intergroup comparisons were made using the Chi-square test or Fisher’s exact test. Sample size was calculated based on a two-sided significance level of 0.05 and power of 80%, assuming a reduction in ICU length of stay from 5.0 ± 2.5 days to 3.5 ± 2.5 days, yielding a minimum of 87 patients per group. Accounting for 10% potential dropouts, we enrolled 141 patients in the NICOM group and 101 in the control group. To address potential confounding and multiple testing, additional analyses were performed. First, for skewed continuous outcomes (ICU length of stay and hospitalization time), quantile regression was used to estimate the median difference between groups, adjusting for age, sex, operative time, cardiopulmonary bypass time, and baseline left ventricular ejection fraction. Second, for binary complications (reintubation, ICU re-admission, shock, and acute heart failure), multivariable logistic regression was applied with the same adjustment variables to calculate adjusted odds ratio and 95% confidence intervals. Third, to control for Type I error inflation due to multiple comparisons (22 secondary endpoints), Bonferroni correction was applied, setting the significance threshold at *p* < 0.0023 (0.05/22). Results with *p* values between 0.0023 and 0.05 were considered nominally significant and interpreted as exploratory. Sensitivity analysis using the Benjamini-Hochberg false discovery rate (FDR) method (q < 0.05) was also conducted to assess the robustness of the findings. A two-sided *p*-value of <0.05 was considered statistically significant for primary and unadjusted analyses, unless otherwise specified.

## 3. Results

### 3.1 Baseline Characteristics

A total of 242 patients were enrolled in this study, with a mean age of 54.9 ± 10.4 years; 66.1% were male. Of these, 141 patients received postoperative NICOM (Group A), while 101 patients did not (Group B) (Fig. 1). The baseline characteristics and preoperative routine laboratory findings of the patients are summarized in Table 1. No significant differences were observed between the two groups at baseline.

**Table 1.** **Comparison of baseline data between the two patient groups**.

Variables	Group A (n = 141)	Group B (n = 101)	*p* value
Male, n (%)	92 (65.2)	68 (67.3)	0.736
Age, (year)	55.8 ± 10.0	53.7 ± 10.5	0.114
Body mass index (kg/m^2^)	25.1 ± 3.6	25.4 ± 3.9	0.668
Hypertension, n (%)	102 (72.3)	79 (78.2)	0.299
Diabetes, n (%)	9 (6.4)	8 (7.9)	0.644
Coronary heart disease, n (%)	14 (9.9)	12 (11.9)	0.629
Marfan syndrome, n (%)	4 (2.8)	7 (6.9)	0.232
History of cardiac surgery, n (%)	7 (5.0)	8 (7.9)	0.347
Leukocyte (10^9^/L)	11.8 ± 4.0	12.6 ± 4.3	0.131
Platelet (10^9^/L)	190.0 (156.0–226.0)	190.0 (155.0–224.0)	0.784
Hemoglobin (g/L)	126.4 ± 21.9	130.0 ± 24.1	0.228
Prothrombin time (sec)	13.8 ± 3.0	14.3 ± 2.3	0.084
D-dimer(µg/mL)	8.5 (3.9–14.6)	8.2 (3.6–14.3)	0.753
Alanine aminotransferase (IU/L)	30.0 (21.5–45.0)	34.0 (21.0–56.0)	0.725
Aspartate aminotransferase (IU/L)	25.0 (19.0–48.5)	28.0 (19.0–46.0)	0.507
Albumin (g/L)	37.2 ± 4.9	37.6 ± 5.3	0.540
Serum creatinine (µmol/L)	81.5 (67.5–109.3)	85.0 (69.0–119.0)	0.335
Troponin I (µg/L)	0.03 (0.01–0.12)	0.03 (0.01–0.20)	0.636
NT-proBNP (pg/mL)	278.0 (115.5–641.0)	288.0 (127.0–865.0)	0.628
LVEF (%)	58.4 ± 7.0	59.1 ± 6.9	0.442

NT-proBNP, N-terminal probrain natriuretic peptide; LVEF, left ventricular ejection fraction.

### 3.2 Intraoperative Data

There were no significant differences between the two groups regarding intraoperative parameters, including operative time, CPB time, aortic cross-clamp time, SCP time, and intraoperative blood loss (Table 2). Furthermore, the surgical strategies for aortic root management (Modified valve-sparing aortic root replacement procedure, Bentall, David, Cabrol, and Wheat procedures) and the techniques for total arch replacement with the frozen elephant trunk technique (Sun’s procedure) or with the triple-branched stent graft (triple-branched stent graft procedure) [2] were also comparable between the groups (*p* > 0.05).

**Table 2.** **Intraoperative data on the two patient groups**.

Variables		Group A (n = 141)	Group B (n = 101)	*p* value
Operation method				
	Sun’s procedure, n (%)	31 (22.0)	22 (21.8)	0.970
	Triple-branched stent graft procedure, n (%)	110 (78.0)	79 (78.2)	0.970
Aortic root concomitant procedure				
Modified valve-sparing aortic root replacement procedure, n (%)		57 (40.4)	43 (42.6)	0.738
David procedure, n (%)		11 (7.8)	7 (6.9)	0.799
Bentall procedure, n (%)		29 (20.6)	23 (22.8)	0.680
Cabrol procedure, n (%)		3 (2.1)	3 (3.0)	0.997
Wheat procedure, n (%)		11 (7.8)	9 (8.9)	0.757
Operative time (min)		310.1 ± 72.7	310.6 ± 67.5	0.959
Cardiopulmonary bypass time (min)		152.7 ± 43.9	153.8 ± 47.6	0.853
Aortic cross-clamp time (min)		50.1 ± 27.0	52.7 ± 24.1	0.455
Selective cerebral perfusion time (min)		12.8 ± 4.5	12.6 ± 4.9	0.760
Intraoperative blood loss (mL)		350.0 (300.0–450.0)	400.0 (310.0–420.0)	0.093

Sun’s procedure: Total aortic arch replacement with the frozen elephant trunk technique. Triple-branched stent graft procedure: Total aortic arch replacement with the triple-branched stent graft.

### 3.3 Postoperative NICOM Parameters in ATAAD Patients

Among the 141 patients monitored with NICOM, the data revealed that 109 (77.3%) exhibited decreased ventricular wall compliance and diastolic dysfunction. Volume overload was present in 25 patients (17.7%). The systemic circulation status was mildly abnormal in 44 patients (31.2%), moderately abnormal in 12 (8.5%), and severely abnormal in 10 (7.1%). Hemodynamic profiling based on NICOM identified 21 patients (14.9%) in a high-output-low-resistance state, 16 (11.3%) in a low-output-high-resistance state, and 3 (2.1%) in a high-output-high-resistance state. No patients (0.0%) were in a low-output-low-resistance state. Isolated high-output, low-output, high-resistance, and low-resistance states were observed in 9 (6.4%), 3 (2.1%), 13 (9.2%), and 1 (0.7%) patient(s), respectively. The detailed parameters are presented in Table 3.

**Table 3.** **Postoperative NICOM data of patients with ATAAD**.

Characteristic		Values (n = 141)
Heart rate (bpm)		93.0 (77.5–107.0)
Stroke volume (mL)		61.8 (52.1–80.9)
Stroke volume index (mL/m^2^)		34.9 (30.1–43.7)
Cardiac output (L/min)		6.1 ± 2.1
Cardiac index (L/min/m^2^)		3.4 ± 1.0
Cardiac contractility index (L/min/m^2^)		73.8 (40.6–105.8)
Thoracic fluid content (KΩ^-1^)		37.0 (32.0–45.0)
Systemic vascular resistance index (dyn·s/cm^-5^·m^2^)		2123.0 (1849.0–2433.0)
Systemic vascular resistance (dyn·s/cm^-5^)		1220.0 (1050–1374.5)
Mean arterial blood pressure (mmHg)		93.0 ± 10.3
Left ventricular ejection time (ms)		215.0 (163.9–293.9)
Early diastolic filling rate (%)		79.1 (60.9–93.4)
Left cardiac work index (kg·m/m^2^)		4.2 ± 1.5
End-diastolic volume (mL)		159.6 (133.3–196.2)
Ejection fraction (%)		51.2 (42.5–63.3)
Decreased ventricular wall compliance, n (%)		109 (77.3)
Decreased diastolic function, n (%)		109 (77.3)
	Fluid overload, n (%)	25 (17.7)
	Mild abnormality in blood circulation, n (%)	44 (31.2)
	Moderate abnormality in blood circulation, n (%)	12 (8.5)
	Severe abnormality in blood circulation, n (%)	10 (7.1)
High-output and low-resistance, n (%)		21 (14.9)
High-output and high-resistance, n (%)		3 (2.1)
Low-output and high-resistance, n (%)		16 (11.3)
Low-output and low-resistance, n (%)		0 (0.0)
High-output and normal resistance, n (%)		9 (6.4)
Low-output and normal resistance, n (%)		3 (2.1)
Normal output and high-resistance, n (%)		13 (9.2)
Normal output and low-resistance, n (%)		1 (0.7)

ATAAD, Acute type A aortic dissection; NICOM, Non-invasive cardiac output monitoring.

### 3.4 Postoperative Mortality and Morbidity

The postoperative mortality rate was comparable between the two groups. However, Group A demonstrated significantly shorter ICU length of stay [3.0 (2.0, 5.0) vs. 5.0 (3.0, 8.0) days, *p* < 0.001] and hospitalization time [16.0 (11.0–21.0) vs. 19.0 (14.0–23.0) days, *p* = 0.001] compared to Group B (Table 4). The rates of reintubation (1.4% vs. 7.9%, *p* = 0.029) and ICU re-admission (2.1% vs. 8.9%, *p* = 0.036) were significantly lower in Group A than in Group B. Furthermore, the incidence of shock (0.7% vs. 5.9%, *p* = 0.045) and acute heart failure (2.1% vs. 9.9%, *p* = 0.018) during hospitalization was significantly lower in Group A. The occurrence of other major complications, including acute kidney injury, gastrointestinal bleeding, cerebral hemorrhage, cerebral infarction, ARDS, sepsis, and MODS, was similar between the groups. Notably, the performance in the 6-minute walk test (6MWT) conducted prior to discharge was significantly better in Group A than in Group B [488.0 (433.0–532.0) vs. 457.0 (410.0–521.0) meters, *p* = 0.02].

**Table 4.** **Postoperative data on the two patient groups**.

Variables	Group A (n = 141)	Group B (n = 101)	*p* value
30-day mortality, n (%)	6 (4.3)	8 (7.9)	0.228
Mechanical ventilation time (h)	38.0 (20.0–73.5)	55.0 (24.0–87.0)	0.123
Reintubation, n (%)	2 (1.4)	8 (7.9)	0.029
ICU length of stay (d)	3.0 (2.0–5.0)	5.0 (3.0–8.0)	<0.001
ICU re-admission, n (%)	3 (2.1)	9 (8.9)	0.036
Hospitalization time (d)	16.0 (11.0–21.0)	19.0 (14.0–23.0)	0.001
6MWT before discharge (m)	488.0 (433.0–532.0)	457.0 (410.0–521.0)	0.020
Acute kidney injury, n (%)	15 (10.6)	10 (9.9)	0.853
ARDS, n (%)	18 (12.8)	17 (16.8)	0.375
Acute heart failure, n (%)	3 (2.1)	10 (9.9)	0.018
Shock, n (%)	1 (0.7)	6 (5.9)	0.045
Gastrointestinal hemorrhage, n (%)	5 (3.5)	6 (5.9)	0.378
Sepsis, n (%)	10 (7.1)	7 (6.9)	0.961
Cerebral hemorrhage, n (%)	2 (1.4)	3 (3.0)	0.705
Cerebral infarction, n (%)	2 (1.4)	2 (2.0)	0.862
Spinal cord injury, n (%)	1 (0.7)	1 (1.0)	0.630
MODS, n (%)	4 (2.8)	5 (5.0)	0.608

ICU, Intensive care unit; ARDS, Acute respiratory distress syndrome; 6MWT, 6-minute walk test; MODS, Multiple organ dysfunction syndrome. For multivariable adjusted and multiple testing results, see **Supplementary Table 1**.

### 3.5 Adjusted and Multiple-Testing Analyses

To address potential confounding and multiple comparisons, we performed additional multivariable analyses as described in the Statistical Analysis section. Quantile regression confirmed that the NICOM group remained independently associated with shorter ICU stay (median difference: –1.8 days; 95% confidence interval (CI): –2.5 to –1.1; *p* < 0.001) and shorter hospitalization time (median difference: –2.9 days; 95% CI: –4.6 to –1.2; *p* = 0.001) after adjusting for age, sex, operative time, cardiopulmonary bypass time, and baseline LVEF. Multivariable logistic regression for binary complications also showed significantly lower odds in the NICOM group for reintubation (adjusted odds ratio (aOR) = 0.16; 95% CI: 0.03–0.78; *p* = 0.023), ICU re-admission (aOR = 0.21; 95% CI: 0.05–0.85; *p* = 0.028), shock (aOR = 0.11; 95% CI: 0.01–0.97; *p* = 0.027), and acute heart failure (aOR = 0.19; 95% CI: 0.05–0.74; *p* = 0.016).

When Bonferroni correction was applied for the 22 secondary endpoints (α’ = 0.0023), only ICU stay and hospitalization time remained statistically significant (both *p* ≤ 0.001). The *p*-values for reintubation, ICU re-admission, shock, and acute heart failure no longer met the corrected threshold and are therefore considered nominally significant. However, Benjamini-Hochberg FDR analysis (q < 0.05) supported the robustness of reintubation (q = 0.041), ICU re-admission (q = 0.045), and acute heart failure (q = 0.041), as well as shock (q = 0.044). These results are summarized in **Supplementary Table 1**.

## 4. Discussion

This comparative study of 242 ATAAD patients undergoing total arch replacement demonstrated that the application of NICOM was associated with significantly superior outcomes in Group A compared to Group B. These improvements included shorter ICU and hospital lengths of stay, as well as lower rates of reintubation and ICU readmission. Furthermore, the better performance in the 6MWT in Group A suggests enhanced cardiopulmonary function and overall recovery.

The primary advantage of NICOM lies in its non-invasive, continuous, and real-time monitoring capability, which allows for the timely detection of changes in patients’ hemodynamic status. This provides clinicians with critical information for guiding fluid management, optimizing the use of vasoactive agents, and assessing cardiac function, thereby supporting individualized treatment strategies [12,13]. The findings of this study further demonstrate that postoperative management guided by NICOM technology enables accurate assessment of ventricular wall motion, identification of reduced ventricular compliance and diastolic dysfunction, and evaluation of volume status and circulatory function. Early detection of abnormalities facilitates timely intervention, effectively stabilizing circulatory function and reducing complications associated with hemodynamic fluctuations or inadequate volume control [14], including acute heart failure, shock, low cardiac output syndrome, and insufficient organ perfusion. Furthermore, this approach lowers the incidence of ICU readmission or reintubation, thereby promoting enhanced postoperative recovery.

In the NICOM group, a hemodynamic pattern characterized by high output and low resistance was observed in 14.9% of patients. This pattern often indicates a tendency toward distributive shock in the early postoperative period and may be associated with systemic inflammatory response syndrome or vasoplegic syndrome [15]. Without timely identification, relative insufficiency in effective circulating volume may lead to reduced organ perfusion pressure, representing a critical precursor to overt shock. Early detection via NICOM supports the judicious use of vasopressors, thereby stabilizing blood pressure and preventing progression to shock and subsequent multi-organ dysfunction [16,17]. Conversely, a pattern of low output and high resistance directly reflects impaired cardiac pump function accompanied by compensatory peripheral vasoconstriction which is a classic hemodynamic profile of acute cardiogenic shock or low cardiac output syndrome [11]. In this study, the significantly lower incidence of acute heart failure in the NICOM group can be attributed to the early identification of patients exhibiting this low-output, high-resistance state. Upon detection of this pattern, timely initiation or adjustment of inotropic therapy, often combined with vasodilators, can improve cardiac function and reduce afterload, thereby preventing the onset of acute heart failure [18].

It is noteworthy that in this study, the proportion of patients with decreased ventricular diastolic function after surgery in the NICOM group was as high as 77.3%, and 17.7% of patients exhibited fluid overload. Following ATAAD surgery, patients often experience decreased ventricular compliance due to myocardial edema and ischemia-reperfusion injury. NICOM can sensitively indicate fluid overload by monitoring relevant derivative parameters [11]. In the presence of diastolic dysfunction, fluid overload significantly increases the risk of pulmonary edema, leading to hypoxemia and increased work of breathing. This is precisely one of the primary reasons for postoperative ARDS and respiratory failure requiring reintubation [19]. In this study, the reintubation rate was lower in the NICOM group, which can be attributed to the fact that precise fluid management effectively supported the smooth recovery of respiratory function. By disrupting the vicious cycle from hemodynamic instability to organ dysfunction, the application of NICOM substantially reduced the incidence of complications such as shock, acute heart failure, and respiratory failure, decreased the rate of ICU readmission due to disease recurrence, and ultimately accelerated patients’ overall recovery findings that are reflected in better 6MWT results.

This study showed no significant differences between the two groups in terms of postoperative mortality, acute kidney injury, cerebral infarction, cerebral hemorrhage, or MODS. Our analysis suggests that in patients following ATAAD, most of these complications represent end-organ manifestations primarily driven by ischemia-reperfusion injury, embolic events, or irreversible cellular damage. The core value of NICOM technology likely lies in its role in perioperative dynamic management, specifically in stabilizing the internal environment and preventing secondary injury [20]. The development of end-organ complications is often dominated by factors such as inadequate cerebral perfusion during intraoperative hypothermic circulatory arrest, thrombotic or air embolism, renal ischemic time, or pre-existing pathological conditions particularly poor organ perfusion whose progression is relatively less modifiable by postoperative hemodynamic management [21,22].

NICOM is primarily used to provide evidence-based support for continuous hemodynamic optimization during the early postoperative period. By enabling real-time identification and early correction of physiological abnormalities, NICOM-guided management more precisely maintains effective organ perfusion pressure and cardiac output, thereby preventing secondary and cumulative organ dysfunction resulting from sustained hypoperfusion or congestion [23]. Through optimization of cardiac function and fluid balance, secondary pulmonary edema and respiratory failure were effectively prevented, further explaining the significantly reduced rate of reintubation [19,24]. This successful prevention of secondary injury constitutes the pathophysiological basis for earlier discharge from intensive care, early mobilization, and ultimately better functional recovery. The differences observed in 6MWT results not only reflect cardiopulmonary reserve but also serve as a comprehensive indicator that multiple organ systems have avoided severe secondary insults and have been able to recover in a coordinated manner. Thus, by enhancing the quality of perioperative hemodynamic management and enabling early quantifiable predictability, the application of NICOM, while not reversing established major organ damage significantly preserves organ functional reserve, thereby laying a solid physiological foundation for the implementation of ERAS protocols. Therefore, in selecting NICOM for this ERAS protocol, we prioritized its non-invasive nature, which avoids the risks of arterial cannulation or nasogastric probe placement. NICOM provides continuous, real-time hemodynamic data without the need for specialized line maintenance, aligning with the ERAS goal of early catheter removal and mobilization. Nevertheless, comparative studies between NICOM and PICCO or oesophageal Doppler in the ATAAD setting are warranted.

This study has several limitations. Its single-center, retrospective design and limited sample size necessitate caution in interpreting the results. Moreover, given the large number of secondary endpoints tested (n = 22), Bonferroni correction indicated that the differences in complication rates (reintubation, ICU re-admission, shock, and acute heart failure) did not remain statistically significant after adjustment for multiple comparisons; therefore, these findings should be interpreted as exploratory and warrant confirmation in future hypothesis-driven studies. Nevertheless, the consistent direction of effect across multiple endpoints and the supportive FDR analysis suggest a genuine signal that merits further investigation. Future multi-center, large-sample, prospective randomized controlled trials are warranted to further validate the efficacy of NICOM in the postoperative management of ATAAD. Additionally, comparative studies evaluating NICOM against other hemodynamic monitoring modalities are warranted.

## 5. Conclusion

Integrating NICOM into the postoperative care of ATAAD patients provides reliable, continuous hemodynamic data that facilitates precise, individualized management. This approach stabilizes circulation, reduces key complications, accelerates recovery, and improves perioperative outcomes, underscoring its vital role in implementing ERAS protocols in cardiac surgery and holds considerable promise for broad clinical adoption.

## Data Availability

The datasets used and analyzed during the current study are available from the corresponding authors on reasonable request.

## Abbreviations

ATAAD, acute type A aortic dissection; ARDS, acute respiratory distress syndrome; aOR, adjusted odds ratio; CTA, computed tomography angiography; CPB, cardiopulmonary bypass; ERAS, enhanced recovery after surgery; FDR, false discovery rate; ICU, intensive care unit; LVEF, left ventricular ejection fraction; MODS, multiple organ dysfunction syndrome; NICOM, non-invasive cardiac output monitoring; SCP, selective cerebral perfusion; 6MWT, 6-minute walk test; PICCO, pulse index continuous cardiac output.
