# Supplementary material for: Application of Non-Invasive Cardiac Output Monitoring in Enhanced Recovery After Surgery for Patients With Acute Type A Aortic Dissection
Source: Rev Cardiovasc Med. 2026 Sep 14;27(9):52101. doi: 10.31083/RCM52101 (PMC13653185; doi:10.31083/RCM52101)
Supplement: Supplementary file 1 [file 2153-8174-27-9-52101-s1.zip › Supplementary Table 1.docx]

**Supplementary Table 1. Multivariable-Adjusted and Multiple-Testing Results for Key Outcomes**

| **Outcome** | Unadjusted P value^*^ | Adjusted effect estimate (95% CI)^†^ | Adjusted P value^‡^ | Bonferroni-corrected significance (P < 0.0023) | FDR q value^§^ |
| --- | --- | --- | --- | --- | --- |
| ICU length of stay (days) | <0.001 | Median difference: -1.8 (-2.5 to -1.1) | <0.001 | Yes | – |
| Hospitalization time (days) | 0.001 | Median difference: -2.9 (-4.6 to -1.2) | 0.001 | Yes | – |
| Re-intubation | 0.029 | aOR: 0.16 (0.03 to 0.78) | 0.023 | No | 0.041 |
| ICU re-admission | 0.036 | aOR: 0.21 (0.05 to 0.85) | 0.028 | No | 0.045 |
| Shock | 0.045 | aOR: 0.11 (0.01 to 0.97) | 0.027 | No | 0.044 |
| Acute heart failure | 0.018 | aOR: 0.19 (0.05 to 0.74) | 0.016 | No | 0.041 |

ICU: intensive care unit; aOR: adjusted odds ratio; CI: confidence interval; FDR: false discovery rate.

* Unadjusted P values derived from Mann–Whitney U test (continuous outcomes) or Chi‑square/Fisher’s exact test (binary outcomes), as originally presented in Table 4.*

†For ICU length of stay and hospitalization time: median difference (NICOM vs. Non-NICOM) estimated by quantile regression adjusting for age, sex, operative time, cardiopulmonary bypass time, and baseline left ventricular ejection fraction (LVEF). For binary outcomes: aOR (NICOM vs. Non-NICOM) from multivariable logistic regression with the same adjustment variables.

‡ Adjusted P values from the respective regression models.

§ Benjamini–Hochberg FDR correction applied across 22 secondary endpoints; q < 0.05 considered significant to control for false discovery rate.

Bonferroni correction was applied to the 22 secondary endpoints, setting the significance threshold at α = 0.05/22 ≈ 0.0023. Only ICU length of stay and hospitalization time remained statistically significant after Bonferroni correction. The other outcomes are considered nominally significant and should be interpreted as exploratory, pending confirmation in larger prospective studies. FDR analysis (q < 0.05) supported the robustness of re‑intubation, ICU re‑admission, and acute heart failure and shock achieve significance by this method.
